# Preliminary Evidence of Endotoxin Tolerance in Dairy Cows during the Transition Period

**DOI:** 10.3390/genes12111801

**Published:** 2021-11-16

**Authors:** Joel Filipe, Alessia Inglesi, Massimo Amadori, Flavia Guarneri, Laura Menchetti, Giulio Curone, Gabriele Brecchia, Daniele Vigo, Federica Riva

**Affiliations:** 1 Dipartimento di Medicina Veterinaria (DIMEVET), Università degli Studi di Milano, 26900 Lodi, Italy; joel.soares@unimi.it (J.F.); giulio.curone@unimi.it (G.C.); gabriele.brecchia@unimi.it (G.B.); daniele.vigo@unimi.it (D.V.); federica.riva@unimi.it (F.R.); 2 Laboratorio Benessere Animale, Biochimica Clinica e Immunologia Veterinaria, IZSLER, 25124 Brescia, Italy; m_amadori@fastwebnet.it (M.A.); flavia.guarneri@izsler.it (F.G.); 3 Dipartimento di Scienze e Tecnologie Agro-Alimentari (DISTAL), University of Bologna, 40126 Bologna, Italy; laura.menchetti@unibo.it

**Keywords:** cattle, PBMC, endotoxin tolerance, transition period, blastogenic response

## Abstract

The blastogenic response of bovine peripheral blood mononuclear cells (PBMCs) to lipopolysaccharides (LPS) has been investigated for a long time in our laboratories. In particular, a possible correlation between the blastogenic response to LPS and the disease resistance of dairy cows has been suggested in previous studies. Isolated PBMCs from eight cows at three different time points during the transition period (T0 = 15 days before calving; T1 = 7 days post-calving; T2 = 21 days post-calving) were cultured in the presence or absence of LPS, and the blastogenic response was assayed 72 h after in vitro stimulation. Moreover, the gene expression of proinflammatory cytokines and kynurenine pathway molecules was investigated by real-time RT-PCR on both unstimulated and stimulated PBMCs. The cows were retrospectively divided into healthy and diseased, based on the development of peripartum diseases (subclinical ketosis and placenta retention). The comparison between healthy and diseased cows suggested that healthy animals seemed to better control the response to LPS. On the contrary, diseased animals showed a much higher inflammatory response to LPS. Moreover, cows were retrospectively classified as high and low responders based on the in vitro proliferative response of PBMCs to LPS, using the median value as a threshold. Unstimulated PBMCs of low responders showed higher expression of the proinflammatory cytokines Interleukin 1-β (IL-1β), Interleukin 6 (IL-6) and Tumor Necrosis Factor-α (TNF-α), compared to high responders. Our preliminary data suggest that, during the peripartum period, high responders seem to be more tolerant to endotoxins and develop a lower inflammatory response to different stressors. Instead, low responders could be more prone to the development of unwanted inflammatory conditions in response to mild/moderate stressors.

## 1. Introduction

Peripheral blood mononuclear cells (PBMCs) include monocytes, dendritic cells and lymphocytes (T cells, B cells, NK cells). These cells can undergo proliferation after stimulation with specific molecules called mitogens, which are classified based on their activity on a specific leukocyte population or different leukocyte populations [1]. LPS is a known mitogen for B and T cells in humans, but only for B cells in mice [2]. In cattle, LPS was demonstrated to act as a mitogen for both B cells and T cells, mainly CD4^+^ T cells [3]. Previous studies suggested a negative correlation between the blastogenic response of PBMCs to LPS and the risk of developing peripartum diseases in dairy cows, differentiating animals into high and low responders, based on the PBMC blastogenic response to LPS [4]. It is well known that, during the transition period, dairy cows show a much higher prevalence of metabolic and infectious diseases [5]. Such diseases take place in the framework of a poorly controlled inflammatory response to diverse infectious and non-infectious stressors around calving, when dramatic physiological and metabolic changes lead to a negative energy balance, mobilization of body reserves and immunodepression [6]. In agreement with these findings, monocytes of Holstein Friesian cows show a peculiar, in vitro response to bacterial products, characterized by elevated inflammasome activation with high levels of IL-1β production, and little if any reactive nitrogen species release, leading to a lower killing capacity of bacteria [7]. The molecular mechanisms at the basis of this peculiar response of high and low responders is still poorly defined. We hypothesized that the kynurenine pathway could be involved. The kynurenine pathway, conserved among species, is the most important means of the metabolic degradation of tryptophan (trp), an essential amino acid [8,9]. The metabolites of the kynurenine pathway carry out different activities in health and disease, underlying, e.g., immune dysfunction, immune tolerance and central nervous system disorders [9]. Tryptophan 2,3-dioxygenase (TDO2) and indoleamine 2,3-dioxygenase 1 (IDO1) are two enzymes involved in the catabolism of the trp and it is known that they are deeply influenced by cytokines and vice versa [10].

In the present study, we investigated the blastogenic response of PMBCs sampled from dairy cows at three different time points during the transition period. Moreover, the gene expression of cytokines (IL-1β, IL-6, TNF-α) and kynurenine pathway molecules (TDO2 and IDO1) was investigated by real-time RT-PCR on both unstimulated and stimulated PBMCs at all three time points. Owing to the above, our working hypothesis implied that a high blastogenic response of bovine PBMCs to LPS underlay the more effective control of the corresponding inflammatory cytokine response, LPS being in vitro an informative correlate of the complex array of stressors experienced in the periparturient period.

## 2. Materials and Methods

### 2.1. Animals and Samples

We collected samples from 8 multiparous Holstein Friesian cows. All the cows were housed in the same farm in Piacenza (Italy), under the supervision of expert bovine practitioners. Cows were housed in free stall barns and milked 2 times daily in a milking parlor. This study complied with Italian laws on animal experimentation and ethics (Italian Health Ministry authorization n. 628/2016-PR). The average milk production of the farm was 10.6 tons/year per cow, with an average content of 3.31% of protein and 3.78% of fat. Venous blood samples were collected from the tail in heparinized vacutainer tubes from the dairy cows at three different time points: T0 = 15 days before the estimated calving day, T1 = 7 days post-calving and T2 = 21 days post-calving. No further sampling and/or additional animal handling were applied to cows. Blood samples were transported at room temperature and processed within three hours after collection. All the cows were routinely tested on the 5th day in milk for the diagnosis of ketosis or hypoglycemia, measuring the β-hydroxybutyrate (BHB) and glucose blood levels, using the Nova Vet™ Ketone/Glucose Meter (Nova biomedical, Waltham, MA, USA). Based on the glucose concentrations, cows were classified as hypo- (<40 mg/dL), normo- (40–60 mg/dL) or hyperglycemic (>60 mg/dL). The BHB values were used to define the sub-clinical 1.2 to 2.9 mmol/L or clinical ≥3.0 mmol/L ketosis condition [11]. Moreover, temperature and vaginal discharge were daily monitored during the first month post-partum to assess the presence of diseases. Based on clinical evaluation, cows were divided into healthy and diseased groups.

### 2.2. Bovine PBMCs Isolation and In Vitro Proliferation

Peripheral blood mononuclear cells (PBMCs) were isolated from venous blood by density gradient centrifugation, as described previously [3]. Briefly, venous blood diluted in PBS (Sigma, St. Louis, MO, USA) was layered over Ficoll-Paque PLUS medium (APB, Milan, Italy) and centrifuged at 600× *g*, 18 °C for 45 min. The mononuclear cell band was recovered and washed twice in PBS. Residual red blood cells were eliminated by hypotonic shock using redistilled water. Polymorphonuclear cell contamination was assessed by flow cytometry based on forward and side scatter parameters and was lower than 5%. Cell viability was checked using the CTL-LCD^TM^ Live/Dead Cell Counting Kit on an ImmunoSpot S6 Ultimate Reader (CTL, Cleveland, OH, USA). Cells were resuspended at 2 × 10^6^ viable cells/mL in RPMI 1640 medium (ThermoFisher Scientific, Waltam, MA, USA) with a content of 2000 mg/L of glucose, Hepes 25 mM, with 2 mM glutamine, supplemented with 10% heat-inactivated fetal calf serum (FCS), 100 U penicillin, 100 µg streptomycin and 0.25 µg amphotericin B/mL (Sigma).

The assay was carried out as previously described [3]. Briefly, 0.2 mL aliquots of PBMCs at 10^6^ cells/mL were reacted in triplicate in 96-well microtiter plates with LPS (from Escherichia coli O111:B4; Sigma, 20 µg/mL or 1 µg/mL final), or with medium only (control), and incubated at 39 °C in 5% CO_2_. After 48 h, 20 µL/well of 100 µM 5-bromo-2-deoxyuridine (BrdU) in culture medium was added to each well, and cell proliferation was quantified after an additional incubation of 18 h, using the Cell Proliferation ELISA BrDU (colorimetric) kit (Roche, cat. 11647229001), according to the manufacturer’s directions. Triplicate control wells containing the PBMC suspension without LPS or BrdU were used. Values of PBMC proliferation were expressed as the mean optical density (OD) for test wells minus the mean OD for control wells without LPS. Bovine PBMCs were also grown with the potent T-cell-specific mitogen Concanavalin A (ConA; 2.5 µg/mL final) under the same conditions as LPS-stimulated cells; ConA was adopted to evaluate the suitability of PBMC cultures to mount blastogenic responses altogether and, accordingly, to validate the results obtained with LPS. Based on the blastogenic response to LPS (20 μg/mL), cows were retrospectively divided into high and low responder groups. In more detail, the median OD of the BrDU assay was calculated for each time point; the animals with responses higher than the median value were allocated to the high responder group, and the animals with lower responses to the low responder group.

### 2.3. In Vitro LPS Stimulation of Bovine PBMCs and Gene Expression Analysis

PBMCs (5 × 10^6^) were plated in 6-well plates with LPS (from Escherichia coli O111:B4; Sigma, 20 µg/mL or 1 µg/mL final), or with medium only (control), and incubated at 39 °C in 5% CO_2_ for 4 h. After the incubation, the cells in suspension were centrifuged for 6 min at 330× *g* at 4 °C. The supernatant was discarded and cells were lysed in 0.5 mL of TRI Reagent (Sigma-Aldrich, St. Louis, MO, USA); the adherent cells were washed with PBS and then lysed with 0.5 mL of TRI Reagent (Sigma-Aldrich, St. Louis, MO, USA). Both adherent and in-suspension cells were pooled together after lysis (1 mL of TRI Reagent total/well). Uncultured PBMCs (5 × 10^6^) were directly lysed in 1 mL of TRI Reagent (Sigma-Aldrich, St. Louis, MO, USA). All samples were frozen at −80 °C until use.

Total RNA was then extracted using the TRI Reagent (Thermo Fisher Scientific, Waltham, MA USA), following the manufacturer’s directions. The concentration and quality of RNA was determined using a spectrophotometer (BioPhotometer, Eppendorf, Hamburg, Germany) at 260/280 nm wavelength. Total RNA (1 µg) was reverse-transcribed using the High-Capacity cDNA Reverse Transcription Kit (Applied Biosystem, Foster City, CA, USA), according to the manufacturer’s instructions. The cDNA obtained from each sample was used as a template for real-time RT-PCR in an optimized 25 µL reaction volume using Sybr Green chemicals, as previously described [12]. Samples were then used to measure the expression of the following genes by real-time RT-PCR: IL-1β, IL-6, TNF-α, IDO1, TDO2. Glyceraldehyde-3-phosphate dehydrogenase (GAPDH) was investigated as the housekeeping gene. Primer pair sequences are listed in Table 1. The primers were purchased from Invitrogen (Carlsbad, CA, USA). Real-time RT-PCR was carried out in the 7000 Sequence Detection System (Applied Biosystems, Foster City, CA, USA), as previously described [9]. Each sample was amplified by real-time RT-PCR in duplicate. The expression of bovine target genes was normalized using the calculated GAPDH cDNA expression (mean) of the same sample and run. The relative quantification of each gene was calculated with the “delta Ct” method [13]. The value obtained was multiplied by “10,000” in order to obtain the Arbitrary Unit.

### 2.4. Statistical Analysis

Statistical analyses were performed using GraphPad Prism 6 (La Jolla, CA, USA), considering statistically significant values at *p* < 0.05 and tendencies at *p* < 0.1. Shapiro–Wilk tests and diagnostic graphics were used to verify the distribution of data and the equality of variances. Since assumptions for parametric tests were violated, gene expression data were log10 transformed and more robust approaches were chosen. Friedman tests were used to analyze changes over time (3 time points) in each gene and the blastogenic response for each treatment. The expression of target genes, production parameters and blastogenic responses between two experimental groups (healthy vs. diseased or high vs. low responders) were compared by the Mann–Whitney test, while Kruskal–Wallis tests were used to analyze the differences between treatments (ConA, LPS 20 and LPS 1) at each time point. Dunn’s tests were used to perform multiple comparisons. Data are presented as medians and first (Q1) and third quartile (Q3).

Finally, two-tailed rho tests of Spearman (ρ) were used to reveal correlations among target gene expression and all the other parameters.

## 3. Results

### 3.1. Proliferation Response of Bovine PBMCs Is Higher in the Dry Period Than at the End of Transition Period

**Figure 1:**
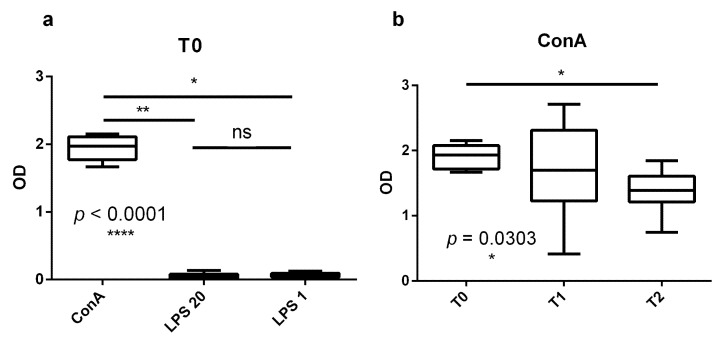
In vitro proliferation of bovine PBMCs at different time points. Bovine PBMCs isolated at three different time points were in vitro stimulated with ConA or LPS (20 μg/mL and 1 μg/mL). Cell proliferation was determined by an ELISA assay after BrDU incorporation. (**a**) Stimulation of PBMCs at 15 days before calving (T0); similar results were obtained at 7 and 21 days post-calving (T1 and T2, respectively). (**b**) Blastogenic response of PBMCs to ConA at three different time points. * *p* < 0.05; ** *p* < 0.01; **** *p* < 0.0001.

As previously observed [3], ConA induced a greater proliferation of bovine PBMCs compared to LPS at both high and low LPS concentrations (20 and 1 μg/mL; *p <* 0.0001) at all three time points (Figure 1a). This confirmed the suitability of our PBMC cultures. Interestingly, the proliferative response to ConA shown by PBMCs at 15 days before calving was statistically higher compared to the response at 21 days post-calving (Figure 1b), presenting a significant time effect (*p* = 0.0303). The proliferation response after LPS stimulation (both at high and low dose) did not show any time effect.

### 3.2. The Expression of Proinflammatory Cytokines Varied during the Transition Period

**Figure 2:**
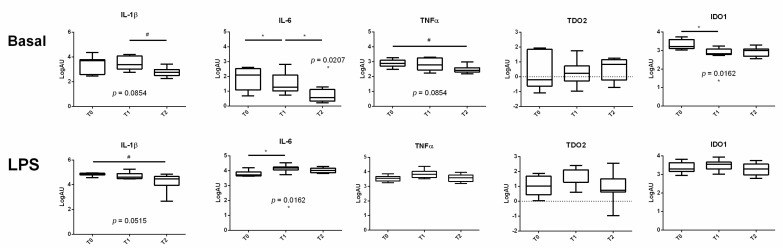
Bovine PBMC gene expression of proinflammatory cytokines and kynurenine molecules at different time points. Bovine PBMCs isolated at 3 different time points (T0 = 15 days before calving; T1 = 7 days post-calving, T2 = 21 days post-calving) were in vitro stimulated with LPS (20 mg/mL) or kept untreated. Gene expression was determined by RT qPCR assay. ^#^ *p* < 0.1, * *p* < 0.05.

The basal (unstimulated) gene expression in PBMCs of proinflammatory cytokines varied at the three time points under study. In particular, IL-6 and IDO1 expression showed a significant time effect (*p* = 0.0207 and *p* = 0.0162, respectively), whereas we only observed a tendency for a time effect in IL-1β and TNF-α expression (both *p* = 0.0854; Figure 2). No time effect was evidenced for TDO2 expression. In more detail, IL-1β expression showed a gradual tendency to decrease from 7 to 21 days post-calving and TNF-α from 15 days before calving to 21 days post-calving. IL-6 expression levels significantly decreased from 15 days before calving to 7 days post-calving and from 7 to 21 days post-calving (Figure 2, upper panels). 

PBMCs stimulated with LPS did not show any time effect on the expression of TNF-α, TDO2 and IDO1, but a tendency in IL-1β and a significant time effect on IL-6 expression (*p* = 0.0515 and *p* = 0.0162, respectively; Figure 2, lower panels). The multiple comparison highlighted moderately decreasing levels of IL-1β from T0 to T2, and a significant increase in IL-6 from 15 days before calving to 7 days post-calving (Figure 2, lower panels). 

### 3.3. Healthy and Diseased Animals Developed Diverging Responses to High Doses of LPS

**Figure 3:**
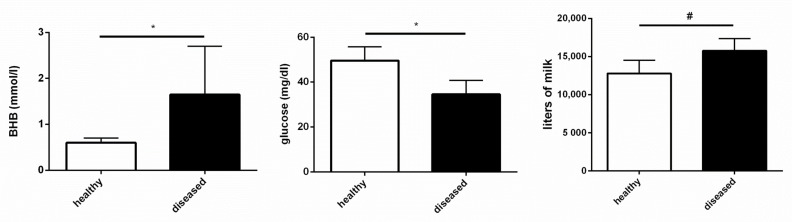
Metabolic and production parameters in healthy and diseased cows. Plasma BHBA and glucose were quantified at 5 DIM (days in milk). Effective milk production was calculated at the end of lactation of each animal. Values are medians, Q1 and Q3. ^#^ *p* < 0.1, * *p* < 0.05.

Four out of the eight cows enrolled in the study encountered peripartum diseases: sub-clinical ketosis (BHB concentration of 1.2 to 2.9 mmol/L) [11] and retained placenta. As expected, diseased animals had higher plasma levels of BHB and lower glycemia (Figure 3); they also showed a greater milk yield (as trend, Figure 3). Accordingly, we observed a significant negative correlation between glycemia and BHB plasma concentration (ρ = −0.814; *p* = 0.0078). Whereas glycemia was negatively correlated (ρ = −0.69; *p* = 0.0395) with the pathology score, BHB was positively correlated (ρ = 0.847; *p* = 0.0079).

**Figure 4:**
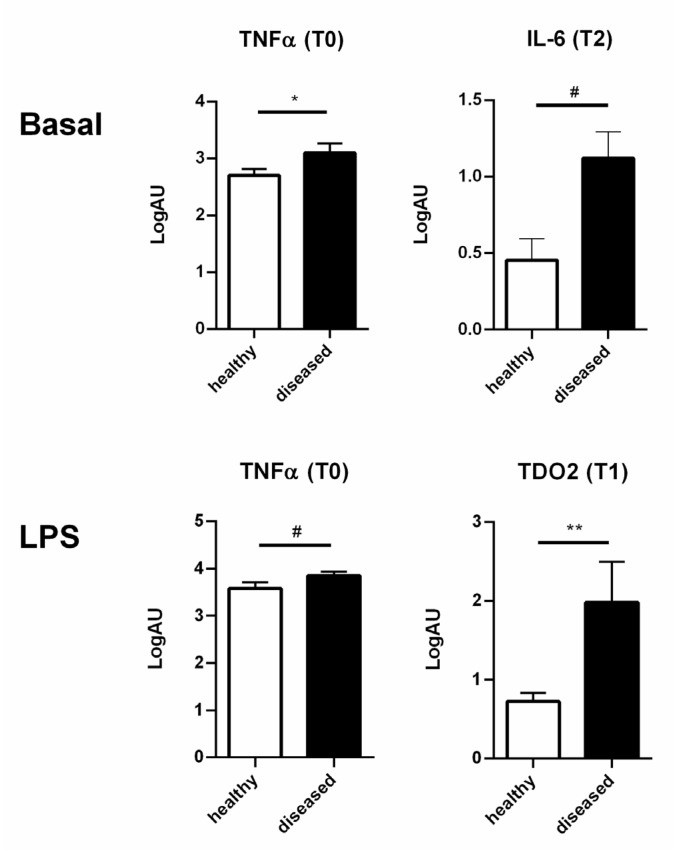
Different responses of PBMCs of healthy and diseased cows. PBMCs of diseased (black bars) and healthy (white bars) cows expressed different levels of proinflammatory cytokines and kynurenin pathway genes under basal conditions and after LPS stimulation at different time points (T0 = 15 days before calving; T1 = 7 days post-calving, T2 = 21 days post-calving). Only the graphs of cytokine expression with *p* < 0.1 or *p* < 0.05 are reported. Values are medians, Q1 and Q3. Gene expression was analyzed by RT qPCR. ^#^ *p* < 0.1, * *p* < 0.05, ** *p* < 0.01.

The comparison between healthy and diseased cows highlighted that healthy animals expressed lower levels of proinflammatory cytokines under basal conditions (TNF-α at 15 days before calving and IL-6 at 21 days post-calving; *p* = 0.0436 and *p* = 0.0631, respectively; Figure 4, upper panels) and showed a tendency towards lower cytokine expression levels after high doses of LPS (20 μg/mL) stimulation (TNF-α at T0; *p* = 0.086; Figure 4, lower panels). On the contrary, diseased animals presented higher expression levels of proinflammatory cytokines under both basal and LPS stimulation conditions (Figure 4). Moreover, PBMCs of diseased cows expressed more TDO2 at 7 days post-calving when compared with healthy animals (*p* = 0.0046). 

### 3.4. High and Low Responders Differently Expressed Cytokine Genes

**Figure 5:**
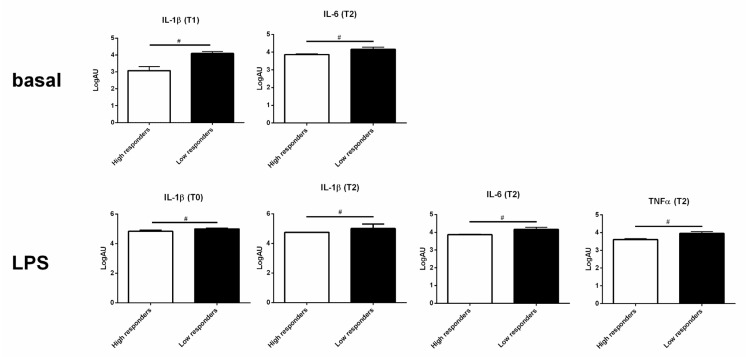
Different responses of PBMCs from high and low responders. PBMCs of high (white bars) and low (black bars) responders expressed different levels of proinflammatory cytokines under basal conditions and after LPS stimulation at different time points (T0 = 15 days before calving; T1 = 7 days post-calving, T2 = 21 days post-calving). Gene expression was analyzed by RT qPCR. Only the graphs of cytokine expression with *p* < 0.1 or *p* < 0.05 are reported. Values are median and Q1 and Q3. ^#^ *p* < 0.1.

Unstimulated PBMCs of low responders showed a tendency towards higher levels of expression of the proinflammatory cytokines IL-1β (*p* = 0.057) and TNF-α (*p* = 0.057) compared to high responders at T1 (Figure 5, upper panels). PBMCs of high responders seemed to better control LPS stimulation at a high dose (20 μg) at 15 days before calving and 21 days post-calving in terms of lower cytokine expression levels (IL-1β at T0; *p* = 0.0643; IL-1β, IL-6 and TNF-α at 21 days post-calving; all *p* = 0.1; Figure 5, lower panels). No differences in the kynurenine pathway gene expression were observed in either unstimulated or stimulated PBMCs at any time point.

### 3.5. Correlations

In order to better understand the molecular mechanisms that could link the proliferative response of PBMCs to their gene expression in vitro, we searched for any possible statistical correlation. In particular, we observed a negative correlation between the proliferation response to LPS at 21 days post-calving and IL-1β and IL-6 gene expression (both ρ = −0.941; *p* < 0.0001). Moreover, IL-6 gene expression after LPS stimulation at 15 days before calving showed a negative correlation with TDO2 gene expression after LPS stimulation at the same time point (ρ = −1.000; *p* = 0.0028). On the other hand, TNF-α gene expression in basal conditions at 21 days post-calving was positively correlated with IDO1 gene expression after LPS stimulation at 15 days before calving (ρ = 1.000; *p* = 0.0028).

## 4. Discussion

Our data confirmed the low proliferative response of bovine PBMCs to LPS compared to ConA [3], adopted in our study as an internal marker. The PBMC blastogenic response to ConA decreased after calving (T2) compared to dry-off (T0); this could be due to increased non-esterified fatty acid (NEFA) plasma levels and the reduced number of CD8^+^ and γ/δ cells after calving [14,15]. Moreover, we observed a different pattern of cytokine expression in unstimulated (basal) and high-dose LPS-stimulated PBMCs. In particular, IL-1β and TNF-α expression seemed to be higher under basal conditions at all three time points compared to IL-6, which seemed to be more active only after LPS stimulation (based on the varying expression of each cytokine under basal conditions and after LPS stimulation at each time point). The different pattern of cytokine expression during the peripartum period was also described previously both in humans and in cattle, as well as the high variability among animals [16,17].

Some of the cows enrolled in the study were affected by diseases during peripartum; as expected, these animals had increased plasma levels of BHB and reduced glycemia compared to healthy cows. This was probably due to a stronger negative energy balance due to the higher milk production of diseased animals. Healthy animals showed lower levels of proinflammatory cytokine gene expression mainly in the dry period (T0) and 3 weeks post-calving (T2), suggesting a better capability to tolerate high doses of LPS. In particular, they could avoid the excessive production of proinflammatory mediators underlying unwanted negative effects. Our results are in agreement with Mezzetti et al. (2019), who highlighted a more pronounced activation of the immune system in terms of higher plasma concentrations of proinflammatory cytokines, myeloperoxidase and oxidant species and higher blood concentrations of γ-glutamyl transferase in subclinical ketosis-affected cows compared to control animals [18].

When cows were divided into high and low responders in terms of the blastogenic response to LPS stimulation, we observed that high responders seemed to better control LPS stimulation at a high dose at 7 and 21 days post-calving in terms of cytokine gene expression levels.

On the whole, the blastogenic and inflammatory responses to LPS were inversely related in our study, in agreement with our working hypothesis. This might be due to a peculiar form of endotoxic tolerance, which involves the kynurenine pathway. The peripartum period is a critical phase for dairy cows, which face dramatic changes in their physiology, nutrition and management; their immune system is deeply involved in these changes, leading to an overt immune dysregulation. Therefore, a deeper knowledge of the molecular mechanisms underlying immune system dysfunction during the peripartum could help to predict the risk of disease development after calving. This might have translational prospects in terms of adequate therapies and management precautions to improve the welfare and productive life of high-yielding dairy cows.

## 5. Conclusions

Given that our data were collected in few animals and samples were collected at three time points, our conclusions should be considered preliminary and further investigations are needed to confirm the above inferences. In particular, our data should be confirmed in a larger cohort of cows. Moreover, it should be interesting to investigate CD180 gene expression, involved in the positive and negative regulation of LPS-driven responses in B cells and macrophages [19]. Finally, the polymorphisms of TLR4 could be investigated in the high and low responders or in the healthy and diseased animals to find a correlation to the LPS response of each dairy cow.

## Figures and Tables

**Table 1:** Primer sequences used for real-time RT-PCR essays.

Gene	Abbreviation	Sequence
Interleukin-1 beta	IL-1β	F: CTG TTA TTT GAG GCT GAT GAC C R: TTG TTG TAG AAC TGG TGA GAA ATC
Tumor Necrosis Factor alpha	TNF-α	F: TCT TCT CAA GCC TCA AGT AAC AAG T R: CCA TGA GGG CAT TGG CAT AC
Interleukin-6	IL-6	F: CAC TCC AGA GAA AAC CGA AGC R: GAA GCA TCC CGT CCT TTT CCT C
Toll-like receptor 4	TLR4	F: CTGCGGCTCTGATCCCAG R: TTAGGAACAACCTGTACGCAAGG
Indoleamine 2,3-dioxygenase	IDO1	F: GGG TCA AGG CGA TGG AGA C R: ACA GCG ATA TTG CTT GGC AA
Tryptophan 2,3-dioxygenase	TDO2	F: TTG AGG CAT GGC TGG AAA G R: AGT TAA ATC CAT GCG GCT CTA AAC
Glyceraldehyde-3-phosphate dehydrogenase	GAPDH	F: GGCGTGAACCACGAGAAGTATAA R: CCCTCCACGATGCCAAAGT

## Data Availability

In this section, please provide details regarding where data supporting reported results can be found, including links to publicly archived datasets analyzed or generated during the study. Please refer to suggested Data Availability Statements in Section “MDPI Research Data Policies” at https://www.mdpi.com/ethics, accessed on 27 September 2021. You might choose to exclude this statement if the study did not report any data.
